# Genome-wide patterns of promoter sharing and co-expression in bovine skeletal muscle

**DOI:** 10.1186/1471-2164-12-23

**Published:** 2011-01-12

**Authors:** Quan Gu, Shivashankar H Nagaraj, Nicholas J Hudson, Brian P Dalrymple, Antonio Reverter

**Affiliations:** 1 Computational and Systems Biology, CSIRO Food Futures Flagship and CSIRO Livestock Industries, 306 Carmody Rd. St. Lucia, Brisbane, Queensland 4067, Australia; 2 College of Information Sciences and Technology, Donghua University, Shanghai 201620, PR China

## Abstract

**Background:**

Gene regulation by transcription factors (TF) is species, tissue and time specific. To better understand how the genetic code controls gene expression in bovine muscle we associated gene expression data from developing *Longissimus thoracis et lumborum *skeletal muscle with bovine promoter sequence information.

**Results:**

We created a highly conserved genome-wide promoter landscape comprising 87,408 interactions relating 333 TFs with their 9,242 predicted target genes (TGs). We discovered that the complete set of predicted TGs share an average of 2.75 predicted TF binding sites (TFBSs) and that the average co-expression between a TF and its predicted TGs is higher than the average co-expression between the same TF and all genes. Conversely, pairs of TFs sharing predicted TGs showed a co-expression correlation higher that pairs of TFs not sharing TGs. Finally, we exploited the co-occurrence of predicted TFBS in the context of muscle-derived functionally-coherent modules including cell cycle, mitochondria, immune system, fat metabolism, muscle/glycolysis, and ribosome. Our findings enabled us to reverse engineer a regulatory network of core processes, and correctly identified the involvement of E2F1, GATA2 and NFKB1 in the regulation of cell cycle, fat, and muscle/glycolysis, respectively.

**Conclusion:**

The pivotal implication of our research is two-fold: (1) there exists a robust genome-wide expression signal between TFs and their predicted TGs in cattle muscle consistent with the extent of promoter sharing; and (2) this signal can be exploited to recover the cellular mechanisms underpinning transcription regulation of muscle structure and development in bovine. Our study represents the first genome-wide report linking tissue specific co-expression to co-regulation in a non-model vertebrate.

## Background

The development of a complex eukaryote originates with just a single cell. A few years later it culminates in a functional organism possessing ~100 trillion cells delineated into ~200 cell types co-ordinately arranged in time and space [1]. A single genome, which is largely static, orchestrates this remarkable event, which is highly dynamic, - but it is still not known how. More specifically, what genomic information turns the right genes on at the right time and in the right place? This problem can be framed in terms of the gene regulation through the action of transcription factors (TFs) on their target genes (TGs) - a well-documented mechanism by which the protein encoded by a TF gene accesses the cell nucleus and binds to TF binding sites (TFBS), located in the promoter region of a TG activating or inhibiting its transcription. Importantly, such regulation acts in a species-, tissue- and time-specific manner [2-4].

Based on such framework, many authors have exploited the guilt-by-association heuristic by which genes regulated by the same TF are more likely to show co-expression correlation and, conversely, sets of genes showing an extreme co-expression correlation are more likely to be regulated by the same set of TFs. Inspired by such heuristic, a rational approach for exploiting this co-expression phenomena and deciphering transcriptional regulation activity involves the reverse-engineering of gene regulatory networks using network inference algorithms such as (but not limited to) Bayesian networks [5], CLR [6]; ARACNE [7], and PCIT [8,9]. Ergün et al. [10] exploited the connectivity structure of a gene network to an expression data set and identified genetic drivers of prostate cancer using the so-called MNI algorithm [11]. Other authors [12-14] have undertaken a promoter sequence analysis of a correlated group of genes to identify sequence motifs corresponding to TFBS. Using the prediction of binding affinities of a TF to promoters, Roider et al. [15] proposed a method for detecting TFs associated with functional categories.

An equally commendable strategy relies on assigning regulators to modules based on the co-expression between a candidate regulator and each of the members of the module. Examples of the latter approach include the LeMoNe (Learning Module Networks) algorithm of Joshi et al. [16] which generates a number of possible models explaining regulation activity and with every single model containing many regulators. An alternative method, initially introduced by Reverter et al. [17] and more recently implemented in Hudson et al. [18], is based on ranking TF by their absolute co-expression correlation averaged across all genes in a given module.

More recently, our group [19] developed a metric, namely RIF for regulatory impact factors, which ranked TFs by analysing the extreme score to those TF that are consistently most differentially co-expressed with the highly abundant and highly differentially expressed genes (RIF1), and to those TF with the most altered ability to predict the abundance of differentially expressed genes (RIF2).

In spite of this plethora of work aimed at identifying the key TFs responsible for a phenotypic contrast of interest (e.g. healthy versus disease), experimental validation linking co-expression with co-regulation remains scarce and, by and large, available only for model organisms. For instance, based on 2,284 yeast genes and 106 TFs, Allocco et al. [20] investigated and quantified the link between co-expression and co-regulation in yeast and concluded that the correlation co-expression must be greater than 0.84 in order for two genes to have a greater than 50% chance of sharing a common TFBS. Also working with 180 yeast TFs, Yu et al. [21] observed that genes targeted by the same TF tend to be co-expressed, with the degree of co-expression increasing if genes share more than one TF. The authors also reported that targets of a given TF tend to have similar cellular functions. Similarly, the relationship between gene co-expression and co-regulation has been explored in *Drosophila melanogaster *[22] and mouse [23]. However, given that only three-fifths of the transcriptional networks are broadly conserved, and the associations between TFs and their TGs are flexible [24], there is a need to explore these matters in the actual organism, and indeed preferably in the tissue of interest, rather than making system-wide predictions based on the closest model organism.

Furthermore, based on the observed relationship between TFs and their TGs, some authors have studied gene regulatory networks for model organisms and focussed on the hierarchical structure of the resulting networks [25,26]. Only more recently, Hu and Gallo [27] provide a catalogue of TF pairs, defined as those with TFBS in the same promoter regions, for human genes and in a tissue-specific manner. However, their work is based on only 214 TF and the authors did not investigate the relationship between TF pairs and co-expression of pairs of genes sharing TF pairs. In the light of these shortcomings, it is apparent that more research is needed to understand the relationship between co-expression and co-regulation.

We have elected to focus our efforts on the development of skeletal muscle and the *Longissimus thoracis et lumborum *(LTL) in particular. Skeletal muscle makes up to 50% of the mass of most mammals and is the single largest tissue contributor to basal metabolism [28]. By merging transcriptional and regulatory information, we aimed to shed light on a range of fundamental hypotheses including: *1) *That a relationship exists between co-expression and co-regulation; *2) *That the way in which TFs partner with each other scales with the number of common TGs and, in turn, influences the amount of co-expression observed between the TFs; and *3) *That TFBS over-represented in the promoter regions of functionally coherent gene modules allows for the identification of TFs and TF networks that are consistent with the biological process of the module.

We first introduce a 'Promoterome' matrix (P-matrix) relating TGs with predicted TFs. In combination with the expression data, this matrix is used to test our first two hypotheses (linking co-expression with co-regulation). We then generate a series of simulated datasets to identify under which conditions, in terms of activation and inhibition, the observed relationship between co-expression and co-regulation can emerge. Finally, we reverse-engineer a network of TFs and discuss its biological relevance in the context of functionally coherent modules.

## Results

### The P-Matrix - A bovine muscle promoterome matrix linking TFs with predicted TGs

We explored the 42,702,661 co-expression correlations among the 9,242 genes of the P-matrix (Additional file 1). Of these, 63% and 37% were positive and negative, respectively. We [18] and others [29-31] have reported a higher reliability for positive correlations, in terms of the ability to replicate them in other datasets, or the results corresponding to validated interactions [32].

**Figure 1:**
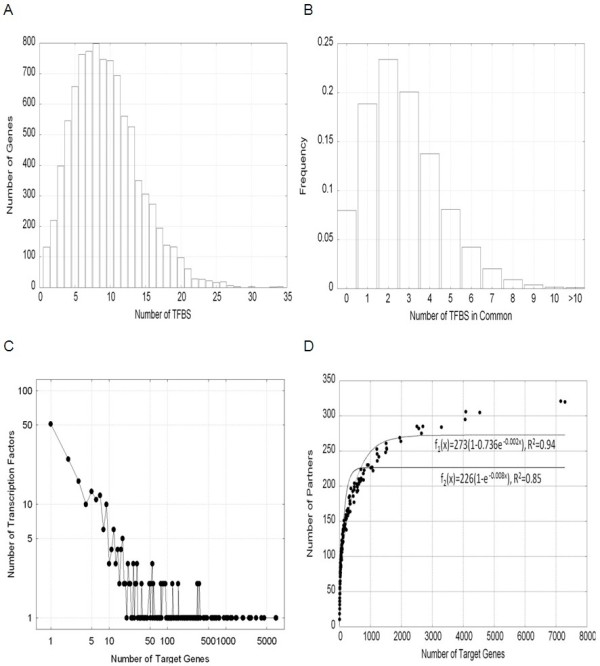
**TFBS data on the Promoterome Matrix (P-matrix)**. (A) Histogram of the number of TFBSs in the promoter region of 9,242 bovine genes; (B) Distribution of the number of common TFBSs in the promoter regions of all gene pairs; (C) Log-log plot of the distribution of TFs as a function of the number of predicted TGs; (D) Number of partners as a function of the number of common TGs for each TF. The best fits for a non-linear growth curve are indicated (i.e. with TF having at least one TG and yielding an R^2 ^of 94%) or forcing the {0,0} co-ordinate (i.e. zero partners if zero TGs and yielding an R^2 ^of 85%).

Figure 1A shows the histogram of the number of TFBS in the promoter region of each of 9,242 genes. 95% of genes have between 3 and 21 TFBS. Figure 1B illustrates the frequency of the number of TFBSs in common for all 42,702,661 gene pairs. 73% of gene pairs have two or more predicted TFBSs in common. Also, 5% of gene pairs share at least 6 TFBSs suggesting these to be components of highly co-regulated networks. Conversely, less than 10% of the gene pairs do not share any TFBSs, suggesting that the vast majority of genes are suitable for co-expression analysis. Figure 1C provides a visual diagnosis of the scale-free behavior of the distribution of TFs as a function of the number of TGs. The number of TGs ranged from 1 (for 48 TFs) to 7,288 (for MZF1; myeloid zinc finger 1) and had a median of 14. Figure 1D reveals an exponential saturation relationship between the number of regulator partners and the number of TGs a given TF has.

### Linking of co-expression, co-regulation and common targets

**Figure 2:**
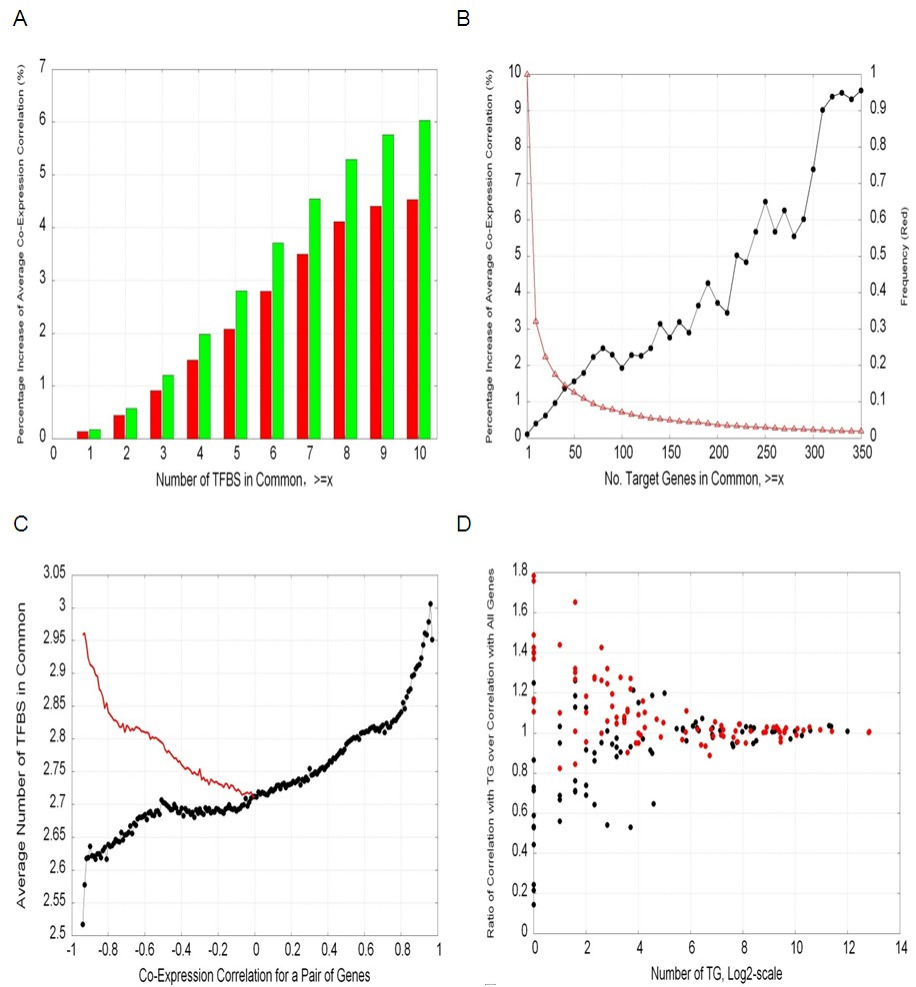
**Linking co-expression and co-regulation**. (A) Percentage increase of co-expression correlations between pairs of TFs as a function of the number of common TFBS and for absolute correlations (red bars) and positive correlations (green bars); (B) Percentage increase (black circles trend) and cumulative frequency (red triangles trend) of co-expression correlations between pairs of TFs as a function of the number of common targets and for absolute correlations; (C) Number of common TFBS as a function of the co-expression correlation for a pair of genes. Observed (black) and naive expectation (red); (D) Target co-expression: Ratio of the average absolute correlation between a TF and its TG over the average absolute correlation between a TF and all genes in the dataset, and as a function of the number of TGs (x-axis). Values on y-axis above one signify the expression of a TF is more correlated with its TGs than with all the genes. Highlighted in red are the 94 TFs for which the average absolute correlation with their TGs is above average.

Figures 2 provide a snapshot of the results relating co-expression with shared TFBSs. We observed an increase in the average co-expression correlation as the number of common TFs increased (Figure 2A). This pattern was consistent irrespective of whether the absolute and positive correlations were considered separately. However, this relationship was more apparent when only positive correlations were considered. For pairs of genes predicted to be jointly targeted by 10 or more TFs, we found a 4% and a 6% increase in absolute and positive correlations respectively, compared with random pairs of genes (Figure 2A).

We also observed (Figure 2B) a strong monotonic increase in the strength of the co-expression between a TF pair and the number of TGs they share. To the best of our knowledge, no study exists exploring this relationship. Among the 15,753 pairs existing from the 178 TFs with expression data, 4,880 where among TF pairs sharing at least one TG. For these, the number of TGs in common averaged 37 and ranged from 1 (for 1,439 TF pairs) to 5,857 (between MZF1 and TGIF1).

One final objective in exploring the relationship between co-expression and co-regulation was to assess whether the average co-expression correlation between a TF and its TGs is higher than the average co-expression between the same TF and all genes in the dataset (Figure 2D). Out of the 178 TFs with expression data, 108 (or 61%) showed a ratio >1, corresponding to a cumulative binomial P-value of 0.001675. When this relationship was limited to the 94 TFs for which their absolute correlation co-expression with their TGs was above average (the average being 0.4334), we found that 71 of them (or 76%) showed a ratio >1 (P-value = 1.13E-07). This distinction is of relevance because, under the null hypothesis of no relationship, TFs showing extreme correlation with their TGs should also show extreme correlation with all genes in the dataset.

### On the superior reliability of positive co-expression correlations

Table 1 provides further evidence that positive correlations are more reliable than negative ones. However, when considering the correlation between a TF and its TGs, and as noted by Yu et al. [33], the sign and magnitude of this correlation is dependant on the regulation type (i.e. activator, repressor or dual) as well as on the regulatory motif (i.e. feed forward, time-shifted, etc.). Therefore, we conclude that using only positive or only negative correlations diminishes the ability to capture true TF - TG relationships and better results emerged when all correlations are used in an absolute context. Importantly, irrespective of which set was used, the evidence becomes more apparent when selecting TF - TG relationships above average. This analysis also showed that as TFs have more TGs the ratio of correlation of the expression of the TFs with its TGs compared to all genes converges to one (Figure 2D).

**Table 1:** Relationship between the expression of a TF and its predicted TG: Ratio of the average correlation between TFs and its TGs over the average correlation between same TF and all the remaining 9,241 genes in the dataset.

	Correlations considered		
	Absolute	Positive	Negative
Ratio >1	108/178 = 60.7%	93/172 = 54.1%	74/149 = 49.7%
P-value	0.001675	0.126331	0.5
Average Correlation (*μ*)	0.433487	0.452847	-0.363314
Ratio >1 and correlation >*μ*	71/94 = 75.5%	66/90 = 73.3%	46/71 = 64.8%
P-value with correlation value >*μ*	1.13E-07	1.9E-06	0.004277

### Simulation analyses reveal the most likely regulation type

**Figure 3:**
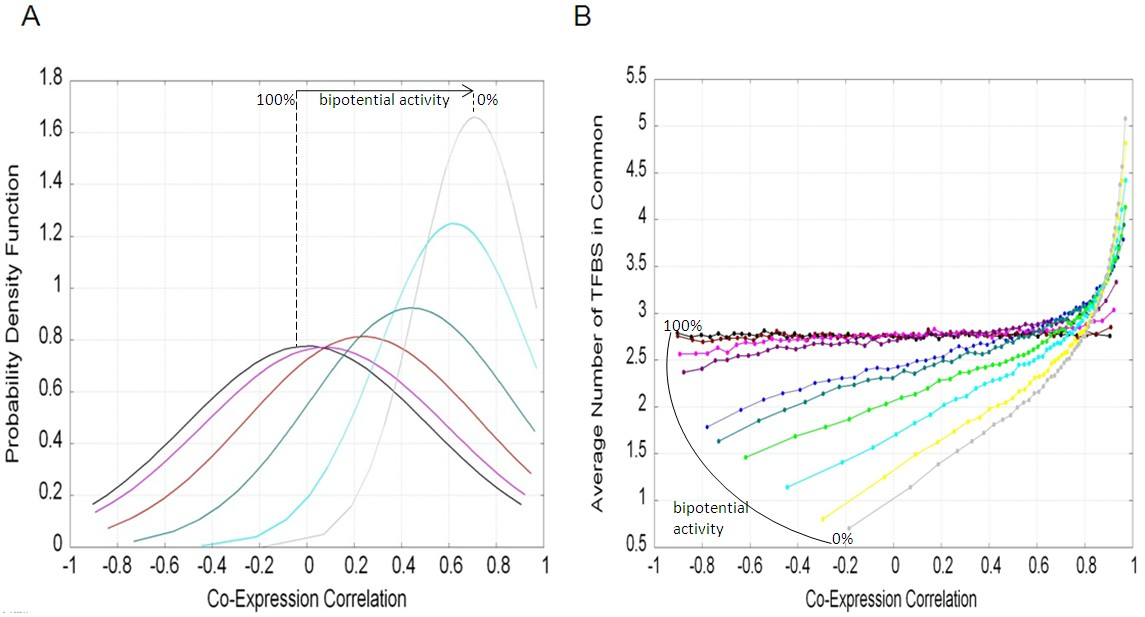
**Simulation result on the linking co-expression and co-regulation**. (A) Distribution of the correlation coefficients as a function of the % of TFs with dual or bipotential activity; (B) Simulation results at varying percentages of transcription factors (TF) operating with bipotential activity: 100% (black), 90% (brown), 80% (pink), 70% (purple), 60% (red), 50% (blue), 40% (jade), 30% (green), 20% (cyan), 10% (yellow), 0% (grey).

As discussed by Yu et al. [21], there are two main reasons for regulation type to impact on the relationship of the expression of their targets. One is that a sizeable proportion of TFs act both as activators and repressors, in some cases for the same target. The other is that the combined effect of multiple TFs can have an unpredictable effect on target expression. Figure 3 illustrates the results from our simulation analyses. One prominent feature is that in order to observe a relationship between co-expression and co-regulation there must be a sizeable proportion of TFs acting as either activators or repressors, but not both. In fact, the relationship between co-expression and co-regulation quickly diminishes with increasing proportion of TFs with bipotential activity (activators and repressors) (Figure 3B). Most interestingly, in the extreme scenario where all TFs have bipotential activity, no relationship between co-expression and co-regulation could be observed and the resulting distribution of the correlations would be perfectly centred at zero (Figure 3A, density shown in black). Instead, such density sees its mass shifted towards the positive space with an increasing proportion of TFs having a single regulation type (Figure 3A, densities shown in colours other than black). We conclude that the higher reliability attributed to positive correlations is a phenomenon of the presence of significant number of TFs that act as either general activators or general repressors and that the co-expression to co-regulation pattern observed from the real expression skeletal muscle dataset is consistent with the presence of 70 to 80% of TFs having a bipotential activity (Figure 2C for real data versus Figure 3B, purple and pink trends, for simulated data). In agreement with our findings, while on a smaller scale, the recent work of Ouyang et al. [34] with mouse embryonic stem cells, revealed that a remarkably high proportion of variation in gene expression can be explained by the binding signals of 12 TFs of which 7 (or 58%) serve as either activator or repressor depending on the target.

### Co-expression as a function of Transcriptional regulatory motifs (TRM)

**Figure 4:**
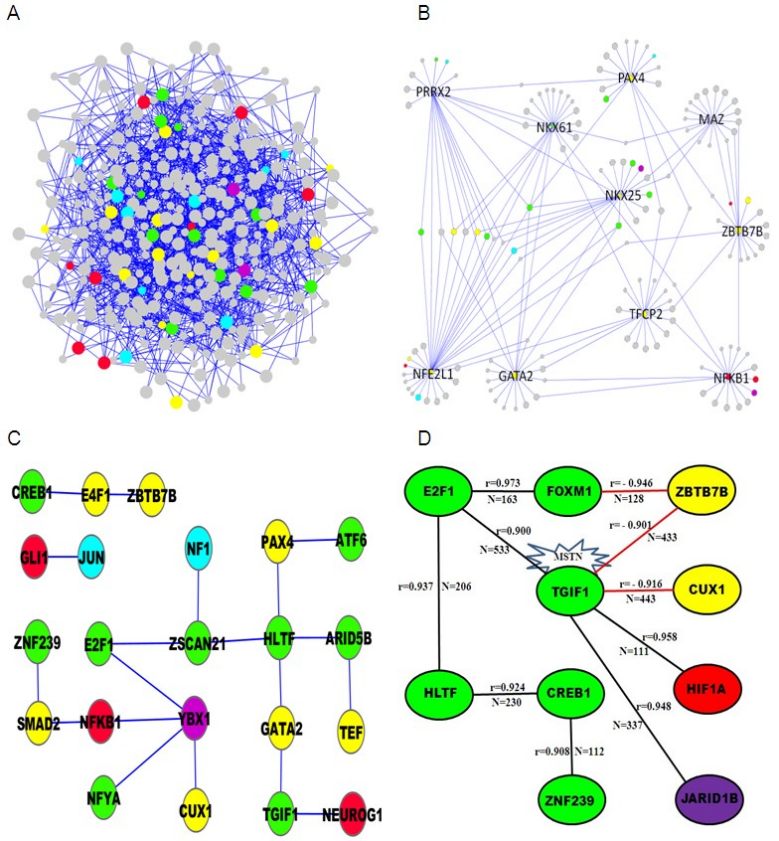
**TF network for bovine skeletal muscle**. (A) Overall view of the 1,395 connections among 333 TFs; (B) Subnetwork spanned by the first neighbors of the 10 most connected TFs (GATA2, MAZ, NFE2L1, NFKB1, NKX25, NKX61, PAX4, PRRX2, TFCP2, and ZBTB7B), details of which are listed in Table 3; (C) Subnetwork of module-specific and muscle-expressed TFs; (D) Network among TFs with absolute correlation (r) >0.9 and number of common TGs (N) >100. Black and red edges correspond to positive and negative correlations, respectively. The network reveals the central role of TGIF1 which also contains a binding motif in the promoter region of myostatin (MSTN). Colors represent functional modules: cell cycle (green), fat (yellow), immune (purple), mitochondria (cyan), and muscle/glycolysis (red). Big and small nodes represent TFs with and without detectable expression in muscle, respectively.

We used the log-odds ratio (LOD) to investigate the relationship between the co-expression correlation observed for genes pairs and the number of shared TFs. We observed that the LOD-value is dependent on the type of transcriptional regulatory motifs (TRM_n_) defining the motif of *n *common TFs jointly regulating the same set of TGs, and with *n *= 0, 1, 2,... up to *n *≥ 10 (Additional File 2). These results corroborated and, to a degree, formally validated our previous observation that gene pairs sharing TFBS showed an increased co-expression correlation (Figure 2A). A similar result was observed for gene pairs sharing from more than 1 to more than 10 TFBSs (Additional File 2). Again, the trend is more pronounced when only positive correlations are considered, in line with a higher reliability for positive correlations [32]. As expected, the distribution of the co-expression correlations for pairs of genes with TRMs of 1, 5 or 10 genes shows an increased bias to highly positive correlations with increased size of the TRM (Figure 4B). Likewise, extreme positive correlations (i.e. within the interval {0.8,1.0}) are more frequent among high-order TRMs than extreme negative correlations (i.e. within the interval {-1.0,-0.8}) (Additional File 2).

### Derivation of a TF network for bovine skeletal muscle

We found 12, 13, 2, 9, 8, and 0 TFs whose TGs were enriched for genes encoding proteins involved in cell cycle, fat, immune, mitochondria, muscle/glycolysis, and the ribosome, respectively (Table 2 and Figure 4C-D). We used the chi-square test of independence to ascertain if there exists an independent pairing assortment in the resulting network of 127 TF and 306 edges. The null hypothesis of independence was rejected (P-value = 2.8359E-62) and we concluded that our module assignment of TFs provided information about the topology of the network.

**Table 2:** Module-specific TFs according to the hypergeometric enrichment test (P < 0.05).

Modules	Number	Transcriptional Factors*
Cell Cycle	12	ARID5B ATF6^** ^CHR **CREB1 E2F1 HLTF NFYA **NKX61 **SP1 TGIF1 ZNF239 ZSCAN21**
Fat	13	**ABL1 CUX1 E4F1 ESRRB GATA2 **IKZF1 MYT1L NKX25 **PAX4 **SOX2 **TEF TFCP2 ZBTB7B**
Immune	2	**ETV4 YBX1**
Mitochondria	9	**CEBPZ **GSH2 **JUN MEF2C NFI NFAT5 **POU3F1 SPZ1 XFD2
Muscle/Glycolysis	8	**GLI1 **HNF4A KLF10 MEIS1** NEUROG1** NFKB1 SMAD2 ZNF300
Ribosome	0	None

*TFs in bold-face correspond to module-specific TFs that are significantly expressed in muscle (Figure 3C).

**ATF6, NEUROG1 and MEIS1 are assigned to different modules depending on the statistical test employed, hypergeometric or odds ratio. For all other TFs, there was a perfect agreement between the two criteria (Table 3).

The TF arrangements were further explored by converting them into a network (Figure 4A). This approach assigned links between TFs predicted to regulate more common targets than expected by chance alone. In order to analyze the resulting network among 333 TF connected by 1,395 edges, we focused on TF hubs in the context of the functionally coherent modules (Figure 4B). In brief, we exploited the muscle-based co-expression modules of Hudson et al. [18] to determine if they represented a robust enough set of TGs to infer the regulation of the biological process in question using promoter data only. To illuminate the relationship between co-expression and the sharing of TGs among TFs, we explored the network that resulted from linking TF pairs with absolute co-expression correlation greater than 0.9 and more than 100 TGs in common. This approach resulted in 10 TFs connected by 10 edges and with TGIF1 as the hub of the network (Figure 4D).

## Discussion

### Co-expression *versus *co-regulation and implications for skeletal muscle

The resulting non-linear pattern observed when relating the number of regulator partners and the number of TGs a given TF has (Figure 1D) resonates with the recent findings of Bhardwaj et al. [35] for model organisms and humans and indicates that only a limited number of partners are required to regulate an increasing number of targets.

As co-expression correlation between a pair of TGs increases, so does the number of common TFs (Figure 2C). Importantly, this relationship continues to trend downwards in negatively co-expressed pairs of genes. Our prior, and in hindsight naïve expectation, was that pairs of genes with extreme negative correlations would share a high number of TFs (overlaid red trend in Figure 2C). Our prior expectation was based on the redundancy mechanism by which one of two genes involved in the same process (and hence regulated by the same TF) suffices at any point in time. However, the observed trend could also be reasoned from a mechanistic standpoint such that in order for a gene pair to show a strong negative co-expression, then their regulators must be vastly different (i.e., these two genes are more likely to be regulated by a completely different set of TFs). To elucidate the most likely speculation, the exploration of various regulation types in the TF - TG relationship in terms of activation, repression or bi-potential, was warranted.

Our analyses also revealed that as TFs have more TGs the ratio of correlation of the expression of the TFs with its TGs compared to all genes converges to one (Figure 2D). This is consistent with the expectation that the more genes a TF regulates, the higher the likelihood that their expression will be also regulated by other TFs. An alternative explanation is that motifs comprised of a single TF regulating many TGs (often refereed to as SIM for single input motifs) can generate temporal programs of expression, in which TGs are activated one by one in a defined order [36,37]. Such temporal order of expression would imply that, at any given time point, the TF - TG relationship could be observed only for some of the TGs.

Pre-eminent among the top 20 TFs with the highest co-expression correlation with their predicted TGs are five HOX genes (HOXB4, HOXC9, HOXA1, HOXC8 and HOXC10). The myogenic regulatory factors (MRF) MYOD1, MYOG, MYF5 and MYF6 are generally not as well-represented as we anticipated, although MYOG is in 18^th ^place. Treating the positive and negative correlations separately improves the representation of the MRF significantly, in that both MYOD1 (17^th ^place) and MYOG (3^rd ^place) subsequently feature. We have previously determined that of the various MRF only MYOD1 and MYOG make it onto an "Always Correlated" network that joins genes exhibiting strong co-expression.

With a special focus on the promoter regions shared by the core muscle structural proteins we determined enrichment for TF predicted to regulate those 66 molecules designated as either "fast", "slow", "embryonic" and "other muscle structural" as listed in Table 2 of Hudson et al.[18]. The strongest enrichment for this process corresponded to gene ontology term "response to retinoic acid", a feature shared by MZF1 (first with 41 promoter hits), TGIF1 (second with 35 hits) and MASH1 (fifth with 22 hits). Other TFs with strong hits included FOXM1 (tenth with 14 hits), a generic cell cycle regulator. The presence of a cell cycle regulator is intriguing given that a key feature of cells progressing through the myogenic program is cell cycle arrest.

There was some evidence for enrichment of the TGF-β signaling pathway, with predicted SMAD2 and SMAD3 binding sites featuring in the upstream regions of a number of targets. MZF1 has been reported to bind to FHL3 (a component of the muscle z-disc) [38], which in turn has been reported to signal via the SMADs [39]. Expression of TGIF1 is induced by TGF-β, has a predicted binding site upstream of MSTN, and interacts with SMAD proteins [40].

### A TF network for bovine skeletal muscle

The P-matrix was subjected to hierarchical cluster analyses. This approach clustered TFs predicted to regulate common TGs, and TGs predicted to be regulated by common TFs. We observed a strong clustering of a set of HOX genes (HOXA1, HOXB13, HOXB4, HOXB9, HOXC1 and HOXD13), which also included the homeobox gene, UNCX. This clustering probably relates to the simple fact that many of the HOX genes are physical neighbors on the genome, formed by relatively recent gene duplication events. In this sense, they are a rare example of physical proximity and shared functionality in eukaryotic genomes [41]. We also observed a cluster of mitochondrial proteins including NDUFA11 near COX6A2 and COXA5, although given the size of the data this may have been expected by chance alone.

### The impact on the functionally coherent modules

The lack of enrichment for the ribosome module is consistent with ribosomal proteins being ubiquitously expressed in all tissues and ribosomal RNA accounting for ~60% of total RNA in the cell [42]. Overall, this approach enriched for some of the known, experimentally-validated regulators, with the cell cycle module performing particularly well. Given our 'promoterome' approach was predicated on first screening for highly conserved cis-regulated modules across mammals, our particular success with the cell cycle may reflect the observation that the cell cycle is more evolutionary conserved than various other processes [43]. For example, the behavior of E2F1, a causal regulator of cell cycle activity [44], recapitulated both here and by high co-expression alone is conserved across groups including *C. elegans *and yeast.

In addition, amongst the module-specific and muscle-expressed TFs (bold-type in Table 2) we highlight ARID5B and FOXM1, both with cell cycle genes significantly enriched among their targets. ARID5B is a member of the AT-rich interaction domain family of TFs, which are known to be critically involved in the regulation of development and cellular differentiation [45]. Recent studies have reported a functional role for FOXM1 in cell cycle [46] and for NFKB1 in muscle [47]. Interestingly, FOXM1 expression is decreased after the induction of fibroblast to myofibroblast transdifferentiation by TGF-β [48].

Equally suggestive is the significant enrichment of genes from the mitochondrial module among the lists of predicted TGs for MEF2C. Van Oort et al. [49] showed that a subset of the genes activated by a dominant negative MEF2C construct in mice encoded proteins localized primarily to, or functioning at, the level of mitochondria. In addition, RNAi silencing of MEF2C expression in the cardiac muscle of mice reduced mitochondrial DNA levels [50]. The nuclear factor 1 (NF1) family of TFs includes NFIX which has been found to act as a transcriptional switch from embryonic to fetal skeletal muscle development [51].

Only three TFs (ATF6, MEIS1 and NEUROG1) changed module allocation depending on the criteria used (hypergeometric test or odds ratio; Table 2 vs Table 3). ATF6 was allocated to cell cycle according to the hypergeometric test and to the fat module according to the odds ratio. ATF6 is a master regulator of the unfolded regulator response and has been implicated the regulation of lipid metabolism [52]. However, in this work the regulation was via a protein-protein interaction with SREBP2 without DNA binding. Similarly, MEIS1 was allocated to the muscle/glycolysis and to the immune module according to the hypergeometric and the odds ratio tests, respectively. MEIS1 belongs in the family of homeobox genes which play a crucial role in many developmental processes. A search of the literature indentified MEIS1 as a regulator of the immune system [53-55] and myogenesis [56].

**Table 3:** Module-specific transcription factors (TF) according to the odds ratio.

Module	Number of TF	Identity of TF
Cell Cycle	33	ARID5B CHR CREB1 E2F1 ETS1 FOXM1 FOXN1 GATA1 GATA3 GCM1 HBP1 HLTF HOXC9 ILF1 MARE MTBF MYF5 MZF1 NFE2L2 NFYA NKX61 NRF1 PBX_HOXA9 PRDM16 PRRX2 SOX5 SP1 TBX5 TGIF1 USF WT1 ZNF239 ZSCAN21
Fat	36	AARE ABL1 ATF6 CUX1 DLX3 DREAM E4F1 ESRRB EVI1 GATA2 HSF2 IK2 IKZF1 LHX3 MASH1 MAZ MSX MYT1 MYT1L NEUROG1 NFE2L1 NKX25 PAX4 RUNX1 RUNX2 SOX2 STAT TAL1 TEF TFCP2 TFII-IR4 ZBTB7B ZEB1 ZIC2 ZNF148 ZNF384
Immune	9	CDX1 CREL ETV4 JARID1B MEIS1 SIX3 TP53 VMYB YBX1
Mitochondria	22	CEBPZ DLX1 GSH2 HNF3B IKZF3 JUN KAISO LHX9 LMX1B MEF2C MSX1 NF1 NFAT5 POU2F1 POU3F1 RELA SIX SMAD3 SPZ1 XFD2 YY1 ZNF35
Muscle/Glycolysis	24	AREB6 BARX2 FAC1 GFI1 GLI1 HIF1A HNF4A KLF10 KLF13 KLF4 MSX2 MYCN NFE2 NFKB1 NOBOX SF1 SMAD2 SMAD4 SOX9 TTF1 YY2 ZF5 ZFX ZNF300
Ribosome	3	CREB2 MYCMAX ZNF219

GATA-binding protein 2 (GATA2) was among the TFs whose TGs were enriched for the fat module genes. Tong et al. [57] discovered that GATA factors play a key role in adipogenesis by suppressing promoters of adipogenic factors including peroxisome proliferator-activated receptor gamma (PPARG).

While the muscle module did not enrich for the MRFs, the presence of SMAD2 and SMAD4 does implicate the TGF-β pathway, known to regulate muscle mass in mammals. The presence of HIF1A, a hypoxia responsive TF, does seem reasonable in a module that contains many glycolysis genes i.e. an alternative energy conversion pathway used in the absence of enough oxygen availability.

### A network linking the GLI transcription factors with MYF5

**Figure 5:**
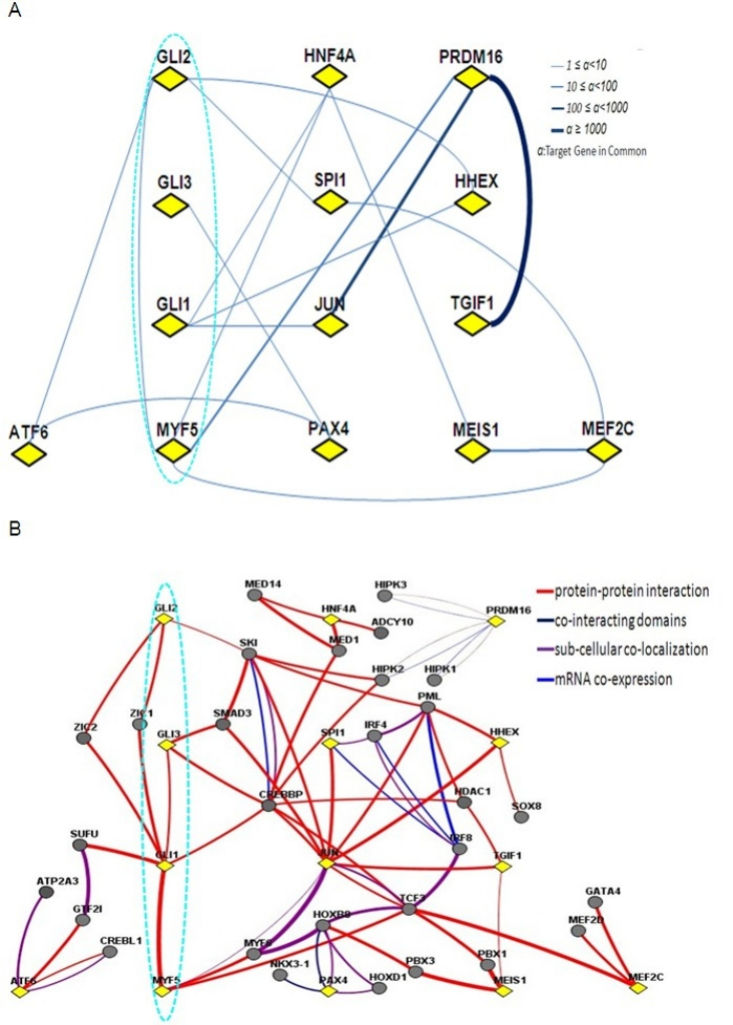
**The network linking the GLI transcription factors with MYF5**. The network linking GLI family and MYF5 (in dashed ellipse) (A) based on our bovine skeletal muscle dataset and (B) the corresponding network based on human datasets according to FUNCOUP tool [59].

An additional correctly inferred regulator of the muscle/glycolysis module genes is GLI1 [58]. Inspired by the work of McDermott et al. [58], who showed that Gli proteins directly control MYF5 expression in mouse muscle progenitor cells, we used our TF network to determine the links between the Gli TFs (GLI1, GLI2 and GLI3), and the myogenic regulatory factor MYF5 (Figure 5A). For comparison, Figure 5B shows the network among the same TFs in human and based on FUNCOUP tool [59]. In our landscape GLI2 was directly linked to MYF5, with one gene in common, and GLI1 was linked to MYF5 via HNF4A. The association of GLI2 and MYF5 in the two datasets are based on quite different types of relationships; GLI2 is required to activate the expression of MYF5 [58], whereas our analysis is based on the predicted co-regulation of target genes by GLI2 and MYF5. The correspondence in the association between MYF5 and GLI2 also provides support that our approach is a useful addition to the toolbox of analysis methodologies to be used in the analysis of gene expression data.

## Conclusions

This work represents the first genome-wide analysis linking tissue-specific co-expression patterns with cis-genomic logic in bovine, a non-model vertebrate. The main finding is that extensive promoter sharing between genes culminates in a detectable and robust co-expression signal, significantly above that which would be expected by chance alone. One pivotal implication is that a sizeable proportion of regulators acting as either activators or repressors, but not both, is needed in order to observe a relationship between co-expression and co-regulation. Finally, we have shown that mining the relationship between promoter sharing and co-expression in the context of functionally coherent modules allows for the identification of key regulators of those modules.

## Methods

### Gene expression dataset

We use the gene expression data from Hudson et al.[18] profiling the genome-wide expression in bovine LTL muscle sampled across 26 experimental conditions as follows: ten developmental time points (3 pre-natal, birth and 6 post-natal) across two beef cattle breed crosses (Piedmontese × Hereford and Wagyu × Hereford) and three time points throughout a nutritional deprivation and re-alimentation experiment comprising 3 adult time points for each of the two treatments (hence, 6 experimental conditions comprised of 3 time points × 2 nutrition treatments). The entire gene expression dataset comprising 48 microarrays has been deposited into Gene Expression Omnibus http://www.ncbi.nlm.nih.gov/geo/ and can be accessed using accession number GSE25554.

Following previously described approaches [60], we fitted the following ANOVA mixed-effect model to normalize the gene expression data:

(1) $${\text{Y}}_{ikvmn}=\mu +{\text{C}}_{ik}+{\text{G}}_{m}+{\text{AG}}_{im}+{\text{DG}}_{km}+{\text{VG}}_{vm}+{\text{e}}_{ikvmn}$$

where Y_*ikvmn *_represents the *n*-th background-adjusted, base-2 log-intensity from the *m*-th gene (*m *= 1, 2, ..., 13094 probes), at the *v*-th experimental condition variety (*v *= 1, 2, ..., 26) taken from the *i*-th array (*i *= 1, 2, ..., 48 microarray chips), and *k*-th dye channel; μ is the overall mean; C represents a comparison group fixed effect defined as those intensities that originate from the same microarray slide, printing block and dye channel; G represents the random gene (or probe) effects with 13,094 levels; AG, DG, and VG are the random interaction effects of array × gene, dye × gene, and variety × gene, respectively; and e is the random error term. Using standard stochastic assumptions, the effects of G, AG, DG, VG and e were assumed to follow a normal distribution with zero mean and between-gene, between-gene within-array, between-gene within-dye, between-gene within-variety and within-gene components of variance, respectively. Restricted maximum likelihood estimates of variance components and solutions to model effects were obtained using VCE6 software ftp://ftp.tzv.fal.de/pub/vce6/. The solutions to the VG effect were used as the normalized mean expression of each gene (or probe) in each of the 26 experimental conditions under scrutiny.

### Promoter sequence analysis

The genome-wide promoter sequence data for bovine was downloaded from Genomatix database http://www.genomatix.de/ (ElDorado Btau 4, v-07-09). A total of 60,131 promoter sequences derived from 22,050 genes were downloaded. We introduced several filtering steps to ensure only high confidence promoter sequences are selected for the analysis. First, we applied the concept of orthologous promoters [61] and retained only those promoter sequences for which phylogenetically conserved promoter sequences were documented in the human and mouse genomes. Using this criterion we retained 39,696 promoter sequences distributed over 13,623 genes. In the next step, we applied a threshold of 1 (100% confidence) to core and matrix similarities [62]. These editing criteria resulted in a total of 310,316 high confidence TFBS that were used for integration with the gene expression data.

### The Promoterome Matrix

We built a 'Promoterome' matrix (P-matrix) relating predicted TGs with TFs based on TFBSs located in their promoter regions. The rows of P-matrix correspond to TGs for which expression data is available, and the columns correspond to TF genes retrieved from the Genomatix database. We identified 9,242 genes (rows of P-matrix) whose promoter sequence (or sequences) harbours at least one predicted TFBS corresponding to at least one of the 333 TFs (columns of P-matrix). The element P(*i*,*j*) of P-matrix is set to "1" if a promoter region of the *i*-th TG contains a TFBS corresponding to the *j*-th TF, otherwise is set to "0". Among the 333 TFs represented in the columns of P-matrix, there were 178 with expression in the Hudson et al. [18] dataset, including 143 with promoter region information in Genomatix (i.e. these 143 TFs were also represented among the rows of P-matrix). We used PermutMatrix [63] to visualise and analyse the resulting P-matrix (See Additional file 1). In addition, the cross-product matrix resulting from multiplying the P-matrix by its transpose, results in a square and symmetric matrix, P^T^P, of dimension equal to 333 (i.e. the number of TFs). Diagonal values of P^T^P matrix (P^T^P(*j,j*)) correspond to the number of TGs for the *j*-th TF. Off-diagonal values of P^T^P (P^T^P(*j,j'*)) correspond to the number of promoter regions in which the TFBS for the *j*-th and the *j'*-th TF co-occur. Co-occurrence is used to build a network of TFs. The hierarchical tree shows a pattern consistent with the non-random assortment of the connectivity distribution with most TFs having few TGs and few TFs having lots of TGs and consistent with a scale-free power-law distribution [64,65].

### Statistical significance in the co-expression to co-regulation relationship

Following Yu et al. [21], we used the log odds ratio (LOD) to ascertain the enrichment of a particular TF - TG relationship with respect to random expectation for the occurrence of the observed co-expression and according to the following formulae:

(2) $$\text{LOD}=\ln\left[\frac{P(\text{coexpression/regulation})}{P(\text{coexpression})}\right]$$

where *P*(co-expression/regulation) is the probability of gene pairs with certain regulatory relationship (e.g. sharing the same predicted TFBS) showing a specific correlation co-expression; and *P*(co-expression) is the probability of randomly selected gene pairs having the same co-expression correlation. LOD values above zero signify observations that are more common than expected by chance, and vice versa.

### Simulation schema

In order to gain further insights into the mechanistic rules governing the observed relationship between co-expression and co-regulation, we generated a series of simulated datasets under various scenarios. Source code in MATLAB (The MathWorks, Inc.) was developed for the simulation schema. In our simulation process a given TF was allowed to act as "dual" (i.e., acting as both activator and repressor of its TGs) or "mono" (i.e., either activator or repressor of its TGs, but not both). In detail, our simulation process followed the following schema:

**Step1**: Define the input vector *D*(*k*) representing the percentage of TFs with "dual" behavior. In detail, *D*(*k*) = (*k*-1) × 10, for *k *= 1, 2, ..., 11. That is, *D *was allowed to range from 0% to 100%, by 10% increment.

**Step2**: At the *r-*th iteration (for *r *= 1 to 93), select a total 100 TGs from P-matrix (i.e., 100 rows of P). Selection is at random and without replacement, ensuring with *r *= 93 that all rows of P were sampled.

**Step3**: Randomly assign the 333 TFs from the P-matrix as follows: *D*(*k*) percentage of TFs are set with dual characteristic while the rest are set with 4 "mono" behaviors in equal amounts: activator, repressor, strong activator, strong repressor.

**Step4**: For the regulation mechanism, allow 5 values of activation/repression as follows: *V *= {-6,-3,0,+3,+6}, where 0 indicates the (lack of) impact of a TF to a non-TG, ± 3 for moderate activator (+3) or moderate repressor (-3), and ± 6 for strong activator (+6) or strong repressor (-6) regulation. These values correspond to either one (± 3) or two (± 6) standard deviation units (see next).

**Step5**: Simulate the expression data at 334 time points corresponding to Time 0 and up to Time 333 for each consecutive TF. In order to approximate the distributional properties of the real data, the expression data for the 100 TGs at Time 0 was simulated at random from a normal distribution with a mean of 9 and a variance of 9 (i.e. *N*(9,9)), and truncated at 0 and 18, as lower and upper bounds, respectively.

At Time *t *(*t *= 1, 2, ..., 333), the *j*-th TF (where *j *= *t*; hence as many time points as TFs) comes into action and the expression of the *i*-th TG is be modified according to the value of *V*. At Time 333, each of the 100 TGs has expression data at 334 dimensions (or time points).

**Step6**: Return to Step2 until the *k *percentage levels and all 9,242 TGs from P-matrix have been explored.

During the simulation process, we also considered the situation in which TFs came into action in groups of five (i.e. having 20 groups from the 100 TFs considered in each iteration). However, the results did not differ from those obtained using the one-at-a-time scenario described above.

### TF network

The strength of the similarity existing for a given TF pair can be inferred from the number of common TGs. We used the columns of P-matrix as the input for the PCIT algorithm [8] to generate a network of TFs. The resulting network contained 333 nodes (i.e. as many as TFs) linked by 1,395 edges. Edges in the network link TFs predicted to share a significantly large number of targets. Consistent with a non-random assortment in the connectivity distribution, the 10 most connected TFs (i.e. ~3% of the total 333 TFs), referred to as 'hub' TFs, had at least 17 connections each, and were connected to 196 TFs (i.e. ~60% of the total).

### Gene modules and significance of TF to module assortment

The functional modules that emerged in the landscape of Hudson et al. [18]were subjected to further scrutiny to generate a curated list of module genes (Additional file 3). This additional examination was based on gene proximity in the hierarchical cluster analysis according to the PermutMatrix software [63] as well as on the molecular function gene ontology term http://www.geneontology.org.

We focused our attention on six modules as follows: cell cycle, fat, immune, mitochondria, muscle/glycolysis, and the ribosome. These modules were chosen for their likely roles in the determination of muscle mass, intramuscular fat development and energetic efficiency.

The enrichment of the affinity between the TFs and the functionally coherent modules was explored by means of the hypergeometric test of significance. To this respect, for every TF - Module combination, we calculated the probability of having the observed number of module genes among its targets using the following hypergeometric equation:

(3) $$H(j,k)=\frac{\left(\begin{array}{c} m_{k}\\ m_{j} \end{array}\right)\left(\begin{array}{c} N-m_{k}\\ n_{j}-m_{j} \end{array}\right)}{\left(\begin{array}{c} N\\ n_{j} \end{array}\right)}$$

where *N *is the total number of genes (9,242), *n*_*j *_is the total number of TGs for the *j*-th TF, *m*_*k *_is the number of genes in the *k*-th module and *m*_*j *_is the number of TG of the *j*-th TF belonging to the *k*-th module.

Those TFs with H(*j,k*) <5% and a proportion (m_*j*_/n_*j*_)/(m_*k*_/N) >1 are referred to as 'module-specific' TFs. The second condition represents an odds ratio and was applied to account for low hypergeometric probabilities resulting from under-enrichment (i.e. those TFs with a proportion of module genes among their targets less than the proportion of module genes among all genes). Finally, among 'module-specific' TF, we classify as 'module-specific and expressed' TF to refer to those for which expression data is available. These criteria resulted in 44 'module-specific' TFs of which 32 were 'module-specific and expressed' TFs (Table 2).

The odds ratio criteria resulted in 127 out of 333 TFs being allocated to one of the six modules (Table 3). In order to ascertain if this module assignment of TFs provided information about the topology of the network we tested if there exists an independent pairing assortment in the resulting network of 127 TFs and 306 edges using the chi-square test of independence (P-value = 2.8359E-62).

## Authors' contributions

QG performed the statistical analyses, devised the simulation schema and drafted the manuscript. SHN performed the promoter sequence analyses and drafted the manuscript. NJH and BPD assessed the biological relevance of the results and drafted the manuscript. AR conceived the study, participated in the statistical analyses and drafted the manuscript. All authors read and approved the final manuscript.

## Supplementary Material

Additional file 1 **Figure S1. Partial view of the Promoterome Matrix (P-matrix)**. Partial view of the Promoterome Matrix (P-matrix) with 9,242 TG in rows and 333 TF in columns and where the TFs have been rear-ranged according to a hierarchical clustering. The hierarchical tree shows a pattern consistent with the non-random assortment of the connectivity distribution with most TFs having few TGs and few TFs having lots of TGs and consistent with a scale-free power-law distribution. Click here for file

Additional file 2 **Figure S2. Further observation on the linking between co-expression and co-regulation**. (A) Log odds ratio (LOD) values as a function of type of transcriptional regulatory motifs (TRM) for absolute correlations (red bars) and positive correlations (green bars) from zero transcription factor (TF) in common (TRM0) to 10 TFs in common (TRM10). LOD values above zero indicate observations that are more common than expected by chance, and vice versa; (B) Difference in LOD-value across the extreme intervals: Extreme positive correlations (i.e. in the {0.8,1.0} interval) and more frequent among high-order TRMs than extreme negative correlations (i.e. in the {-1.0,-0.8} interval); (C) LOD-values for the co-expression as a function of the number of TFs in common for absolute correlations (red bars) and positive correlations (green bars); (D) Empirical density distribution of correlations at three TRM from TRM1 (red) to TRM5 (green) to TRM10 (blue). Click here for file

Additional file 3 **Table S1. Module Genes**. Gene content of the six functional modules profiled in this study. Click here for file

## References

[B1] Lewin R Complexity: Life at the Edge of Chaos 2000 Chicago: University of Chicago Press

[B2] Levine M Tjian R Transcription regulation and animal diversity Nature 2003 424 147 151 10.1038/nature01763 12853946

[B3] Wasserman WW Sandelin A Applied bioinformatics for the identification of regulatory elements Nat Rev Genet 2004 5 276 287 10.1038/nrg1315 15131651

[B4] Chen K Rajewsky N The evolution of gene regulation by transcription factors and microRNAs Nat Rev Genet 2007 8 93 103 10.1038/nrg1990 17230196

[B5] Friedman N Linial M Nachman I Pe'er D Using Bayesian networks to analyze expression data J Comput Biol 2000 7 601 620 10.1089/106652700750050961 11108481

[B6] Faith JJ Hayete B Thaden JT Mogno I Wierzbowski J Cottarel G Kasif S Collins JJ Gardner TS Large-scale mapping and validation of Escherichia coli transcriptional regulation from a compendium of expression profiles PLoS Biol 2007 5 e8 10.1371/journal.pbio.0050008 17214507 PMC1764438

[B7] Margolin AA Wang K Lim WK Kustagi M Nemenman I Califano A Reverse engineering cellular networks Nat Protocols 2006 1 662 671 10.1038/nprot.2006.106 17406294

[B8] Reverter A Chan EKF Combining partial correlation and an information theory approach to the reversed engineering of gene co-expression networks Bioinformatics 2008 24 2491 2497 10.1093/bioinformatics/btn482 18784117

[B9] Watson-Haigh NS Kadarmideen HN Reverter A PCIT: an R package for weighted gene co-expression networks based on partial correlation and information theory approaches Bioinformatics 2010 26 411 413 10.1093/bioinformatics/btp674 20007253

[B10] Ergün A Lawrence CA Kohanski MA Brennan TA Collins JJ A network biology approach to prostate cancer Mol Syst Biol 2007 3 82 17299418 10.1038/msb4100125 PMC1828752

[B11] di Bernardo D Thompson MJ Gardner TS Chobot SE Eastwood EL Wojtovich AP Elliott SJ Schaus SE Collins JJ Chemogenomic profiling on a genome-wide scale using reverse-engineered gene networks Nat Biotechnol 2005 23 377 383 10.1038/nbt1075 15765094

[B12] Kerhornou A Guigó R BioMoby web services to support clustering of co-regulated genes based on similarity of promoter configurations Bioinformatics 2007 23 1831 1833 10.1093/bioinformatics/btm252 17496321

[B13] Cowley MJ Cotsapas CJ Williams RBH Chan EKF Pulvers JN Liu MY Luo OJ Nott DJ Little PFR Intra- and inter-individual genetic differences in gene expression Mamm Genome 2009 20 281 295 10.1007/s00335-009-9181-x 19424753 PMC2690833

[B14] Kumar CG Everts RE Loor JJ Lewin HA Functional annotation of novel lineage-specific genes using co-expression and promoter analysis BMC Genomics 2010 11 161 10.1186/1471-2164-11-161 20214810 PMC2848242

[B15] Roider HG Manke T O'Keeffe S Vingron M Haas SA PASTAA: identifying transcription factors associated with sets of co-regulated genes Bioinformatics 2009 25 435 442 10.1093/bioinformatics/btn627 19073590 PMC2642637

[B16] Joshi A De Smet R Marchal K Van de Peer Y Michoel T Module networks revisited: computational assessment and prioritization of model predictions Bioinformatics 2009 25 490 496 10.1093/bioinformatics/btn658 19136553

[B17] Reverter A Hudson NJ Wang Y Tan S Barris W Byrne KA McWilliam SM Bottema CDK Kister A Greenwood PL Harper GS Lehnert SA Dalrymple BP A gene coexpression network for bovine skeletal muscle inferred from microarray data Physiol Genomics 2006 28 76 83 10.1152/physiolgenomics.00105.2006 16985009

[B18] Hudson NJ Reverter A Wang Y Greenwood PL Dalrymple BP Inferring the transcriptional landscape of bovine skeletal muscle by integrating co-expression networks PLoS ONE 2009 4 e7249 10.1371/journal.pone.0007249 19794913 PMC2749936

[B19] Reverter A Hudson NJ Nagaraj SH Pérez-Enciso M Dalrymple BP Regulatory impact factors: unraveling the transcriptional regulation of complex traits from expression data Bioinformatics 2010 26 896 904 10.1093/bioinformatics/btq051 20144946

[B20] Allocco DJ Kohane IS Butte AJ Quantifying the relationship between co-expression, co-regulation and gene function BMC Bioinformatics 2004 5 18 10.1186/1471-2105-5-18 15053845 PMC375525

[B21] Yu H Luscombe NM Qian J Gerstein M Genomic analysis of gene expression relationships in transcriptional regulatory networks Trends Genet 2003 19 422 427 10.1016/S0168-9525(03)00175-6 12902159

[B22] Marco A Konikoff C Karr TL Kumar S Relationship between gene co-expression and sharing of transcription factor binding sites in Drosophila melanogaster Bioinformatics 2009 25 2473 2477 10.1093/bioinformatics/btp462 19633094 PMC2752616

[B23] Kim RS Ji H Wong WH An improved distance measure between the expression profiles linking co-expression and co-regulation in mouse BMC Bioinformatics 2006 7 44 10.1186/1471-2105-7-44 16438730 PMC1403805

[B24] Essien K Stoeckert CJ Conservation and divergence of known apicomplexan transcriptional regulons BMC Genomics 2010 11 147 10.1186/1471-2164-11-147 20199665 PMC2841118

[B25] Yu H Gerstein M Genomic analysis of the hierarchical structure of regulatory networks Proc Natl Acad Sci USA 2006 103 14724 14731 10.1073/pnas.0508637103 17003135 PMC1595419

[B26] Jothi R Balaji S Wuster A Grochow JA Gsponer J Przytycka TM Aravind L Babu MM Genomic analysis reveals a tight link between transcription factor dynamics and regulatory network architecture Mol Syst Biol 2009 5 294 10.1038/msb.2009.52 19690563 PMC2736650

[B27] Hu Z Gallo SM Identification of interacting transcription factors regulating tissue gene expression in human BMC Genomics 2010 11 49 10.1186/1471-2164-11-49 20085649 PMC2822763

[B28] Henriksson J The possible role of skeletal muscle in the adaptation to periods of energy deficiency Eur J Clin Nutr 1990 44 Suppl 1 55 64 2193804

[B29] Lee HK Hsu AK Sajdak J Qin J Pavlidis P Coexpression analysis of human genes across many microarray data sets Genome Res 2004 14 1085 1094 10.1101/gr.1910904 15173114 PMC419787

[B30] Voy BH Scharff JA Perkins AD Saxton AM Borate B Chesler EJ Branstetter LK Langston MA Extracting Gene Networks for Low-Dose Radiation Using Graph Theoretical Algorithms PLoS Comput Biol 2006 2 e89 10.1371/journal.pcbi.0020089 16854212 PMC1513268

[B31] Güell M van Noort V Yus E Chen W Leigh-Bell J Michalodimitrakis K Yamada T Arumugam M Doerks T Kühner S Rode M Suyama M Schmidt S Gavin A Bork P Serrano L Transcriptome complexity in a genome-reduced bacterium Science 2009 326 1268 1271 19965477 10.1126/science.1176951

[B32] Ewing RM Chu P Elisma F Li H Taylor P Climie S McBroom-Cerajewski L Robinson MD O'Connor L Li M Taylor R Dharsee M Ho Y Heilbut A Moore L Zhang S Ornatsky O Bukhman YV Ethier M Sheng Y Vasilescu J Abu-Farha M Lambert J Duewel HS Stewart II Kuehl B Hogue K Colwill K Gladwish K Muskat B Kinach R Adams S Moran MF Morin GB Topaloglou T Figeys D Large-scale mapping of human protein-protein interactions by mass spectrometry Mol Syst Biol 2007 3 89 10.1038/msb4100134 17353931 PMC1847948

[B33] Yu H Greenbaum D Xin Lu H Zhu X Gerstein M Genomic analysis of essentiality within protein networks Trends Genet 2004 20 227 231 10.1016/j.tig.2004.04.008 15145574

[B34] Ouyang Z Zhou Q Wong WH ChIP-Seq of transcription factors predicts absolute and differential gene expression in embryonic stem cells Proc Natl Acad Sci USA 2009 106 21521 21526 10.1073/pnas.0904863106 19995984 PMC2789751

[B35] Bhardwaj N Carson MB Abyzov A Yan K Lu H Gerstein MB Analysis of combinatorial regulation: scaling of partnerships between regulators with the number of governed targets PLoS Comput Biol 2010 6 e1000755 10.1371/journal.pcbi.1000755 20523742 PMC2877725

[B36] Zaslaver A Mayo AE Rosenberg R Bashkin P Sberro H Tsalyuk M Surette MG Alon U Just-in-time transcription program in metabolic pathways Nat Genet 2004 36 486 491 10.1038/ng1348 15107854

[B37] Ronen M Rosenberg R Shraiman BI Alon U Assigning numbers to the arrows: parameterizing a gene regulation network by using accurate expression kinetics Proc Natl Acad Sci USA 2002 99 10555 10560 10.1073/pnas.152046799 12145321 PMC124972

[B38] Takahashi K Matsumoto C Ra C FHL3 negatively regulates human high-affinity IgE receptor beta-chain gene expression by acting as a transcriptional co-repressor of MZF-1 Biochem J 2005 386 191 200 10.1042/BJ20040775 15453830 PMC1134781

[B39] Ding L Wang Z Yan J Yang X Liu A Qiu W Zhu J Han J Zhang H Lin J Cheng L Qin X Niu C Yuan B Wang X Zhu C Zhou Y Li J Song H Huang C Ye Q Human four-and-a-half LIM family members suppress tumor cell growth through a TGF-beta-like signaling pathway J Clin Invest 2009 119 349 361 19139564 10.1172/JCI35930 PMC2631293

[B40] Wotton D Lo RS Lee S Massagué J A Smad transcriptional corepressor Cell 1999 97 29 39 10.1016/S0092-8674(00)80712-6 10199400

[B41] Michalak P Coexpression, coregulation, and cofunctionality of neighboring genes in eukaryotic genomes Genomics 2008 91 243 248 10.1016/j.ygeno.2007.11.002 18082363

[B42] Ide S Miyazaki T Maki H Kobayashi T Abundance of Ribosomal RNA Gene Copies Maintains Genome Integrity Science 2010 327 693 696 10.1126/science.1179044 20133573

[B43] Ettwiller L Budd A Spitz F Wittbrodt J Analysis of mammalian gene batteries reveals both stable ancestral cores and highly dynamic regulatory sequences Genome Biol 2008 9 R172 10.1186/gb-2008-9-12-r172 19087242 PMC2646276

[B44] Qin XQ Barsoum J Differential cell cycle effects induced by E2F1 mutants Oncogene 1997 14 53 62 10.1038/sj.onc.1200809 9010232

[B45] Watanabe M Layne MD Hsieh C Maemura K Gray S Lee M Jain MK Regulation of smooth muscle cell differentiation by AT-rich interaction domain transcription factors Mrf2alpha and Mrf2beta Circ Res 2002 91 382 389 10.1161/01.RES.0000033593.05545.7B 12215486

[B46] Penzo M Massa PE Olivotto E Bianchi F Borzi RM Hanidu A Li X Li J Marcu KB Sustained NF-kappaB activation produces a short-term cell proliferation block in conjunction with repressing effectors of cell cycle progression controlled by E2F or FoxM1 J Cell Physiol 2009 218 215 227 10.1002/jcp.21596 18803232 PMC2581928

[B47] Bakkar N Guttridge DC NF-kappaB signaling: a tale of two pathways in skeletal myogenesis Physiol Rev 2010 90 495 511 10.1152/physrev.00040.2009 20393192

[B48] Untergasser G Gander R Lilg C Lepperdinger G Plas E Berger P Profiling molecular targets of TGF-beta1 in prostate fibroblast-to-myofibroblast transdifferentiation Mech Ageing Dev 2005 126 59 69 10.1016/j.mad.2004.09.023 15610763

[B49] van Oort RJ van Rooij E Bourajjaj M Schimmel J Jansen MA van der Nagel R Doevendans PA Schneider MD van Echteld CJA De Windt LJ MEF2 activates a genetic program promoting chamber dilation and contractile dysfunction in calcineurin-induced heart failure Circulation 2006 114 298 308 10.1161/CIRCULATIONAHA.105.608968 16847152

[B50] Pereira AHM Clemente CFMZ Cardoso AC Theizen TH Rocco SA Judice CC Guido MC Pascoal VDB Lopes-Cendes I Souza JRM Franchini KG MEF2C silencing attenuates load-induced left ventricular hypertrophy by modulating mTOR/S6K pathway in mice PLoS ONE 2009 4 e8472 10.1371/journal.pone.0008472 20041152 PMC2794538

[B51] Messina G Biressi S Monteverde S Magli A Cassano M Perani L Roncaglia E Tagliafico E Starnes L Campbell CE Grossi M Goldhamer DJ Gronostajski RM Cossu G Nfix regulates fetal-specific transcription in developing skeletal muscle Cell 2010 140 554 566 10.1016/j.cell.2010.01.027 20178747

[B52] Zeng L Lu M Mori K Luo S Lee AS Zhu Y Shyy JY ATF6 modulates SREBP2-mediated lipogenesis EMBO J 2004 23 950 958 10.1038/sj.emboj.7600106 14765107 PMC381012

[B53] Wang GG Song J Wang Z Dormann HL Casadio F Li H Luo J Patel DJ Allis CD Haematopoietic malignancies caused by dysregulation of a chromatin-binding PHD finger Nature 2009 459 847 851 10.1038/nature08036 19430464 PMC2697266

[B54] Camargo A Ruano J Fernandez JM Parnell LD Jimenez A Santos-Gonzalez M Marin C Perez-Martinez P Uceda M Lopez-Miranda J Perez-Jimenez F Gene expression changes in mononuclear cells in patients with metabolic syndrome after acute intake of phenol-rich virgin olive oil BMC Genomics 2010 11 253 10.1186/1471-2164-11-253 20406432 PMC2874810

[B55] Kumar AR Sarver AL Wu B Kersey JH Meis1 maintains stemness signature in MLL-AF9 leukemia Blood 2010 115 3642 3643 10.1182/blood-2010-01-264564 20430967 PMC2867272

[B56] Grade CVC Salerno MS Schubert FR Dietrich S Alvares LE An evolutionarily conserved Myostatin proximal promoter/enhancer confers basal levels of transcription and spatial specificity in vivo Dev Genes Evol 2009 219 497 508 10.1007/s00427-009-0312-x 20052486

[B57] Tong Q Tsai J Hotamisligil GS GATA transcription factors and fat cell formation Drug News Perspect 2003 16 585 588 10.1358/dnp.2003.16.9.829340 14702139

[B58] McDermott A Gustafsson M Elsam T Hui C Emerson CP Borycki A Gli2 and Gli3 have redundant and context-dependent function in skeletal muscle formation Development 2005 132 345 357 10.1242/dev.01537 15604102

[B59] Alexeyenko A Sonnhammer ELL Global networks of functional coupling in eukaryotes from comprehensive data integration Genome Res 2009 19 1107 1116 10.1101/gr.087528.108 19246318 PMC2694487

[B60] Reverter A Barris W McWilliam S Byrne KA Wang YH Tan SH Hudson N Dalrymple BP Validation of alternative methods of data normalization in gene co-expression studies Bioinformatics 2005 21 1112 1120 10.1093/bioinformatics/bti124 15564293

[B61] Buske FA Bodén M Bauer DC Bailey TL Assigning roles to DNA regulatory motifs using comparative genomics Bioinformatics 2010 26 860 866 10.1093/bioinformatics/btq049 20147307 PMC2844991

[B62] Cartharius K Frech K Grote K Klocke B Haltmeier M Klingenhoff A Frisch M Bayerlein M Werner T MatInspector and beyond: promoter analysis based on transcription factor binding sites Bioinformatics 2005 21 2933 2942 10.1093/bioinformatics/bti473 15860560

[B63] Caraux G Pinloche S PermutMatrix: a graphical environment to arrange gene expression profiles in optimal linear order Bioinformatics 2005 21 1280 1281 10.1093/bioinformatics/bti141 15546938

[B64] Barabási A Scale-free networks: a decade and beyond Science 2009 325 412 413 19628854 10.1126/science.1173299

[B65] Barabási A Oltvai ZN Network biology: understanding the cell's functional organization Nat Rev Genet 2004 5 101 113 14735121 10.1038/nrg1272

