# Supplementary figures and images for: Genome-wide patterns of promoter sharing and co-expression in bovine skeletal muscle
Source: BMC Genomics. 2011 Jan 12;12:23. doi: 10.1186/1471-2164-12-23 (PMC3025955; doi:10.1186/1471-2164-12-23)

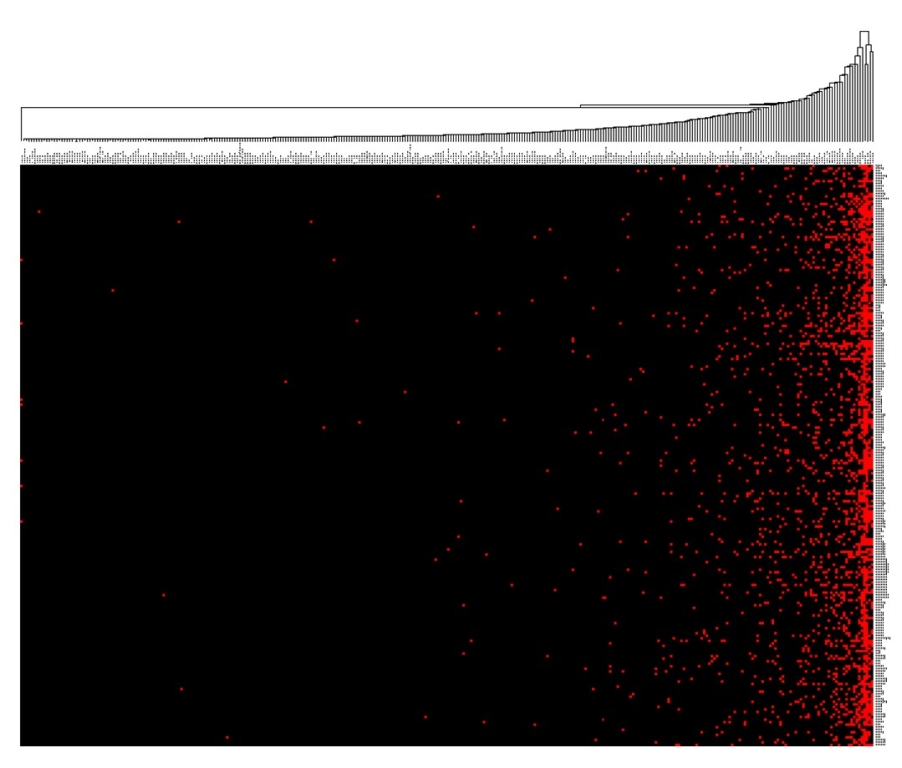

Supplement: Additional file 1 — Figure S1. Partial view of the Promoterome Matrix (P-matrix). Partial view of the Promoterome Matrix (P-matrix) with 9,242 TG in rows and 333 TF in columns and where the TFs have been rear-ranged according to a hierarchical clustering. The hierarchical tree shows a pattern consistent with the non-random assortment of the connectivity distribution with most TFs having few TGs and few TFs having lots of TGs and consistent with a scale-free power-law distribution. [file 1471-2164-12-23-S1.JPEG]

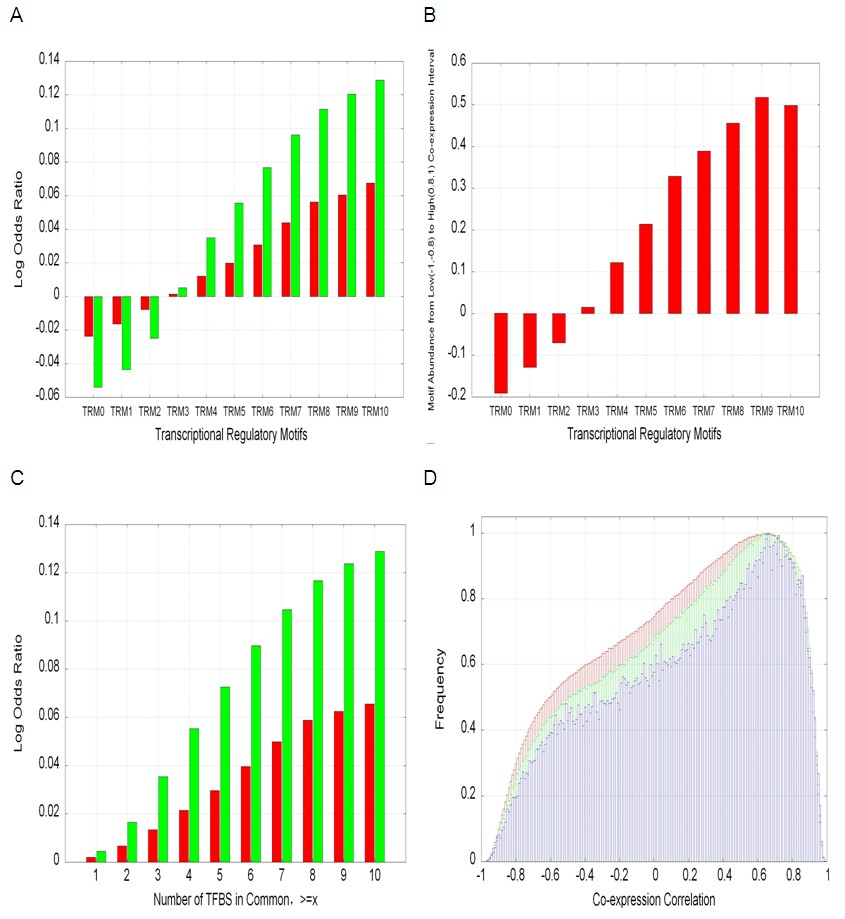

Supplement: Additional file 2 — Figure S2. Further observation on the linking between co-expression and co-regulation. (A) Log odds ratio (LOD) values as a function of type of transcriptional regulatory motifs (TRM) for absolute correlations (red bars) and positive correlations (green bars) from zero transcription factor (TF) in common (TRM0) to 10 TFs in common (TRM10). LOD values above zero indicate observations that are more common than expected by chance, and vice versa; (B) Difference in LOD-value across the extreme intervals: Extreme positive correlations (i.e. in the {0.8,1.0} interval) and more frequent among high-order TRMs than extreme negative correlations (i.e. in the {-1.0,-0.8} interval); (C) LOD-values for the co-expression as a function of the number of TFs in common for absolute correlations (red bars) and positive correlations (green bars); (D) Empirical density distribution of correlations at three TRM from TRM1 (red) to TRM5 (green) to TRM10 (blue). [file 1471-2164-12-23-S2.JPEG]
