# Supplementary material for: Genome-wide patterns of promoter sharing and co-expression in bovine skeletal muscle
Source: BMC Genomics. 2011 Jan 12;12:23. doi: 10.1186/1471-2164-12-23 (PMC3025955; doi:10.1186/1471-2164-12-23)
Supplement: Additional file 3 — Table S1. Module Genes. Gene content of the six functional modules profiled in this study. [file 1471-2164-12-23-S3.DOC]

| Module | Number | Genes Symbol |
| --- | --- | --- |
| Cell Cycle | 49 | ANLN CCDC99 CCNA2 CCNB1 CCNB2 CCNF CDC2 CDC20 CDC25A CDC25B CDC6 CDCA2 CDCA3 CDCA5 CDCA8 CDKN3 CENPE CENPF CEP55 CHEK1 CKAP2 DLGAP5 E2F1 E2F2 E2F8 FOXM1 KCNQ1 KIAA0101 KIF15 KIF20A KIF23 KIF4A KIFC1 MND1 NDC80 PAPSS1 PBK PLK4 PRR11 PTTG1 RASSF4 RRM2 SMC2 SPC24 SPC25 TACC3 TOP2A TROAP UHRF1 |
| Fat | 19 | ACSM1 ACSS2 ADIG ADIPOR1 ADIPOR2 AGPAT2 CIDEA CIDEC DGAT2 FABP4 FADS1 FADS2 FBP1 LDLR PCK1 PIP5K1B PLIN PLS1 TUSC5 |
| Immune | 22 | ASPA BOLA-DMB BOLA-DRA BSG C1R CD22 CD244 CD3E CD3G CD74 CTSW FCGR3A HLA-DRA IKZF3 IL2RB IRF1 IRF9 ITGAL LCP1 MYO1G PSMB8 PSMB9 |
| Mitochondria | 33 | ACO2 APOO BRP44 COQ9 COX5A COX7A1 COX7B CYCS DLAT DLD ECHDC3 ECSIT ENDOG ES1 GBAS GOT2 MDH1 MDH2 MRPS36 NDUFA3 NDUFA5 NDUFB5 NDUFS2 NDUFS3 NDUFS7 NDUFV1 NDUFV2 NNT PDHA1 PDHX SLC25A3 SUCLA2 UQCRC1 |
| Muscle / Glycolysis | 44 | ACTN3 AK1 AKR1B10 ATP2A1 BGLAP BIN1 CDH22 CIDEB CKM CMTM4 DHDH DHRS7C ENO3 GPI GRM6 HESX1 HRC IDH3A ITGB4 KCNG2 LAMB3 LZTR1 MACROD1 MTX3 MYBPC2 MYH1 MYLK2 MYOM2 MYOZ1 NEB NT5C2 PAX2 PGAM2 PGM1 PKM2 PYGM SLC16A3 SUV420H2 TBX15 TNNT3 TPI1 TPM1 TPM2 UBXN1 |
| Ribosome | 22 | RPL11 RPL13 RPL13A RPL18 RPL18A RPL19 RPL23 RPL24 RPL35A RPL38 RPL5 RPL8 RPLP0 RPS11 RPS14 RPS15 RPS19 RPS24 RPS3 RPS5 RPS7 RPS9 |
